# SEM Evaluation of Marginal Adaptation E-Max Crowns Manufactured by Printing-Pressed and Milling

**DOI:** 10.3390/diagnostics13233518

**Published:** 2023-11-23

**Authors:** Ana Ispas, Marioara Moldovan, Stanca Cuc, Doina Prodan, Cecilia Bacali, Ioan Petean, Smaranda Buduru, Manuela Manziuc, Laura Iosif

**Affiliations:** 1 Department of Prosthodontics, Faculty of Dental Medicine, “Iuliu Hatieganu” University of Medicine and Pharmacy, 400006 Cluj-Napoca, Romania; 2 Raluca Ripan Institute for Research in Chemistry, Babes-Bolyai University, 30 Fantanele Street, 400294 Cluj-Napoca, Romania; 3 Faculty of Chemistry and Chemical Engineering, Babes-Bolyai University, 11 Arany Janos Street, 400028 Cluj-Napoca, Romania; 4 Department of Prosthodontics, Faculty of Dental Medicine, “Carol Davila” University of Medicine and Pharmacy, 010221 Bucharest, Romania

**Keywords:** E-Max crowns, marginal adaptation, SEM microscopy

## Abstract

Dental crown marginal adaptation is a matter of the success of dental restoration treatment. Nowadays, there are many technological ways for crown manufacturing, such as tridimensional printing of an exactly desired shape through CAD-assisted systems and the appropriate shape milling of a predesigned bulk crown. Both methods are developed for patient benefits. The current research aims to investigate the marginal adaptation of E-Max crowns manufactured by printing-pressed and milling methods. The in vitro cementation procedures were effectuated on healthy teeth extracted for orthodontic purposes according to the standard procedures and the marginal adaptation was investigated with SEM microscopy. The restoration overview was inspected at a magnification of 100× and the microstructural details at 400×. The integrity of marginal adaptation was properly inspected in identical samples on segments of 2 mm from each buccal, palatal, distal and mesial side. The obtained results reveal a good marginal adaptation for all samples, with some particularities. The statistical analysis shows that the best values of the marginal adaptation were obtained for vestibular/buccal and palatal sides of the teeth being situated around 90–95%, while the values obtained for distal and mesial sides are slightly lower such as 80–90%. Furthermore, it was observed that the milled crowns presents better marginal adaptations than the printed-pressed ones, sustained by the statistical *p* < 0.05. This indicates that the milling process allows a better fit of the crown to the tooth surface and preserves the integrity of the bonding cement layer.

## 1. Introduction

Glass ceramic restorations have gained more and more attention in recent decades, mainly due to the patients’ increased demands for esthetic smiles and secondly due to the technological development in the field of dentistry. Rich crystalline ceramic materials were developed for metal–ceramic restoration replacement.

Lithium disilicate crystals (LD), as a widely used glass ceramic material, are inserted into a ceramic matrix consisting of quartz, lithium dioxide, alumina, phosphorus oxide, potassium oxide, and other oxide substitutes [1]. It is known to be one of the best versatile metal-free alternatives among the all-ceramic types, being applied both for anterior and posterior purposes [2]. Thus, the material exhibits excellent mechanical properties, such as a flexural strength of 300–520 MPa [3] and a fracture toughness of 2.0 MPa/m [4], which is much higher compared to the glass ceramics materials from a few years ago [2]. There are two different manufacturing methods for LD crystallization: through milling or through the lost wax hot pressing technique [5]. The base ceramic is obtained through pressure-casting in steel molds for both methods.

However, clinicians are still debating which processing method, either conventional or digital, produces restorations with better marginal accuracy and internal fit [2,6]. Paramount characteristics for the clinical longevity of LD restorations include an aesthetic aspect, high fracture resistance and an acceptable marginal fit [7]. Proper marginal fit and fewer internal gaps ensure an increased survival rate for the future crown.

Generally, morphological disproportions between the teeth and restorations are the cause of these gaps’ appearance [8]. The marginal gap term is described as the space between the preparation line and the crown. Vasiliu et al. described it as the disparity between the edge of the crown and the tooth [8]. On the other hand, inner gaps are the result of spaces between the dental structure and the inner part of the restoration.

An acceptable marginal fit value that can be universally adopted has not been proposed. However, some researchers consider values of around 120 µm to be tolerable [9,10,11]. Other authors claim that it should be less than 100 μm [12,13], but there are still specialists arguing about values ranging between 20–75 µm, such as the American Dental Association [14]. The latter value is rarely found in clinical practice.

In cases of increased marginal discrepancy, the cement becomes thicker under the influence of the oral cavity environment, leading to cement dissolution. As a consequence, tooth biofilm accumulates, favoring the appearance of caries lesions, micro leakage, and pulp infections and ultimately arising to periodontal lesions and bone loss. Moreover, poor internal adaptation determines lower retention and resistance for the tooth-restoration complex. A thicker cement layer triggers a higher concentrated force that can lead to the appearance of micro-cracks and marginal fractures [9].

In order to assess the marginal gap, different methods can be applied. Sorensen et al. categorized them into four main groups: in direct observation, clinical assessment using a dental probe, impression replica method and cross-sectional method [15]. In this regard, the simplest evaluation method is assessing the marginal adaptation by direct observation with a mirror and probe, which mostly relies on the clinicians’ experience and is less accurate due to the influence of subjectivism [7,8]. The silicone replica technique is a popular non-destructive method for evaluating marginal and internal adaptation [16]. In this regard, a light body polyvinylsiloxane material records the space between the inner surface of the restoration and the preparation. The resulting silicone film can be further analyzed at multiple points at the margins and internal walls of the crown. It is considered an inexpensive and accurate method, but on the other hand silicone tearing, few reference points and sectioning in the wrong plane are to be mentioned drawbacks. Another non-invasive technique is represented by micro-CT analysis [8], which usually evaluates two sections for each extracted tooth, the sagittal and coronal [7].

However, one of the most commonly applied procedures is the cross-sectioning method. Here, the restorations and prepared teeth are cut and the evaluation of the space corresponding to the cement gaps is carried out with the Scanning Electron Microscopy (SEM). SEM marginal analysis is currently the most commonly used conservative method with high magnifying power that ensures very accurate measurements of the marginal errors and parameter of fit [17]. Literature data indicates a low magnification of about 100× for the marginal adaptation overview aspect and a magnification of 400–500× for the microstructural details [18,19,20].

Although the technique has some disadvantages, such as the destructive character regarding the sample preparation steps (molding, cutting and polishing), which can lead to damage of the sample [16], SEM remains at the present time one of the most capable methods to provide adequate findings of the marginal and fit variance of fixed partial dentures.

There are two distinct methods for LD restoration production: lost wax hot pressing and 3D printing. The first method is based on high vacuum injection, where ceramic ingots are hot-pressed in a mold where a waxed crown is invested. The second method is represented by three-dimensional printing, also known as additive manufacturing, which is a newly developed technology. This can be performed by replacing the conventional waxed restoration with 3D printed ones by using the pressing technique. The fact is sustained by newest trends indicating CAD-CAM printing as a more effective fabrication technological process of composite parts destined to dental applications [21,22]. Thus, the purpose of our study consisted of comparing the marginal adaptation of LD (E-Max) crowns fabricated via CAD-CAM technology with 3D printing-pressing (3D Asiga Max, Sydney, Australia) and classical milled crowns using SEM. The null hypothesis we intended, therefore, to test was that marginal adaptation among LD dental crowns made by different manufacturing methods exhibits the same accurate fit.

## 2. Materials and Methods

The present in vitro study was developed at the Prosthetic Dentistry Department of “Iuliu Hatieganu” University of Medicine and Pharmacy Cluj-Napoca, Romania. Marginal adaptation of all ceramic crowns was measured by using the direct view technique with a SEM having the support and collaboration of the “Raluca Ripan” Institute of Chemistry and Research, Cluj-Napoca, Romania. The research protocol was approved by the Ethics Committee of “Iuliu Hatieganu” University of Medicine and Pharmacy Cluj-Napoca and was registered under the number 253/13.09.22.

**Figure 1:**
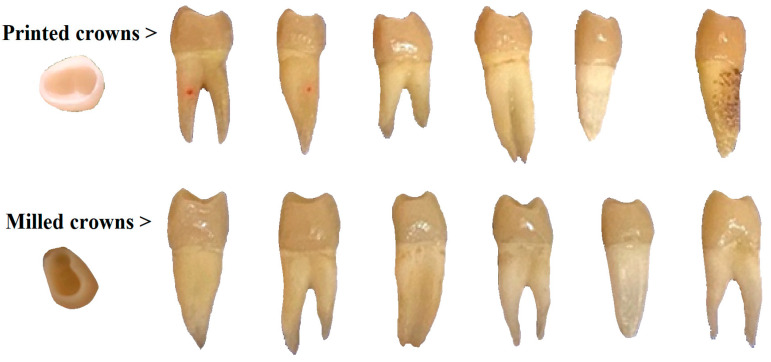
General aspects of the printed and milled crowns and cemented samples on the investigated teeth.

The research included 20 healthy, caries-free human premolars extracted for orthodontic purposes, obtained from private dental cabinets. The teeth were disinfected just after extraction and stored in artificial saliva at 4 °C before tooth preparation. The preparation procedure consisted of making a silicone key in order to have a better grip during preparation by condensation mixing of a silicone elastomer with a high viscosity and consistency, Protesil Putty (Vaninni Dental Industry, Grassina, Italy) and 2 lines of corresponding catalyst material. 20 premolar teeth were further prepared, presenting the following design:

(1) Margin design: 1 mm circumferential shoulder;
(2) 6° convergence of axial wall;
(3) Axial reduction: 1.5 mm;
(4) Occlusal reduction: 1.5–2 mm;
(5) Support lingual cusp beveled (Figure 1).

A cylindrical diamond bur of 2 mm diameter was used for shoulder and axial reduction during the preparation of a blue ring. The occlusal surface was reduced with a blue ring olive-shaped bur: Komet Dental (Gebr. Brasseler GmbH & Co. KG, Lemgo, Germany) No. 00015750. The beveling of the support cusp was realized with a red ring flame diamond bur. A red ring cylindrical diamond (Komet Dental Gebr. Brasseler GmbH & Co. KG, Lemgo, Germany, No. 00051237) and olive bur with a diameter of 2 mm were used for the finishing at a rotational speed of 30,000 rpm with water cooling. After the grinding process, the premolars were kept in numbered containers with artificial saliva. The comparison was done first visually following the crown-tooth interface of the premolars, evaluating their continuity and the presence of gaps and fissures from the mesial, distal, buccal and oral sides.

All teeth were placed in the extra oral 3D Open Tech Smart Scanner (Open Tech 3D, Brescia, Italy) to make the design of ceramic crowns using the EXO CAD (Exocad GmbH, Darmstadt, Germany). The crown design and finish line marking were planned with this software. The parameters were as follows: virtual die spacer: 90 mm, occlusal milling offset: −175 mm, proximal contact strength: 25 mm, occlusal contact strength: −50 mm, radial minimal thickness: 500 mm, occlusal minimal thickness: 1000 mm, and marginal thickness: 50 mm. The crown design was established according to the used materials specifications from the manufacturer; details are presented in Table 1. The success of crown manufacturing and subsequent endodontic restoration depends on the proper adjustment of the design in agreement with the used material prescriptions [23].

The 20 premolars were divided into two groups according to the technological production of the crowns (milling and 3D printed-pressed).

For the CAD/CAM milling group (*n* = 10), the crowns were milled from monolithic LD (IPS E-Max CAD, Ivoclar Vivadent AG, Schaan, Liechtenstein) ceramic blocks using the Imes-Icore CORiTEC milling machine (Eiterfeld, Germany).

In the printed-pressed group (*n* = 10), the crowns were 3D printed (3D Asiga Max Sydney, Australia) using a polymeric material-Burn (Premarket Tasarimve Teknnoloji, Istanbul, Turkey). The 3D printed crowns were invested, and then monolithic LD (IPS E-Max Press Ivoclar Vivadent AG) ingots were pressed in a pressing furnace (Programat EP 3000, Ivoclar Vivadent AG, Schaan, Liechtenstein). The monolithic LD crowns were removed from the investment, and then polished and cleaned from excess material. Before cementation, the crowns were verified on the abutment teeth for their fit. The inner surfaces of the crowns were cleaned with water and air dried. Further Monobond Etch & Prime (Ivoclar Vivadent AG, Schaan, Liechtenstein) was applied for 20 s on the inner surfaces of the crowns, rinsed for 40 s and dried for 10 s.

All the crowns were then cemented on the extracted premolar abutments using the dual-cure luting composite Variolink Esthetic DC (Ivoclar Vivadent AG, Schaan, Liechtenstein), following the manufacturer’s recommendations. The aspect of printed-pressed crown restored teeth is presented in the upper row of Figure 1. Thus, the premolars were first rinsed with water and dried with the air-water syringe. As the next step, the etching gel was applied on the entire abutment surface for 15 s followed by rinsing and drying.

Then universal adhesive was applied on the entire tooth surface for 20 s, dried and polymerized for 10 s. Variolink Esthetic DC (Ivoclar Vivadent AG, Schaan, Liechtenstein) cement from the syringe was applied on the inner surface of the crowns and seated on the abutments with finger pressure until final polymerization. Each surface (buccal, palatal, mesial and distal) was polymerized for 2 s using a Blue Phase Power Cure (Ivoclar Vivadent AG, Schaan, Liechtenstein) at a power of 3000 mW/cm^2^. The excess material was removed with a curette.

Another step was the application of Liquid Strip (Ivoclar Vivadent AG, Schaan, Liechtenstein) from the syringe in the cervical region, followed by polymerization of the aforementioned surfaces for 10 s each. Furthermore, the cervical regions of crowns were polished with a rubber cup. Finally, the marginal adaptation for the two types of differently manufactured crowns was comparatively evaluated. Pictures were therefore taken at different magnifications (100× for restoration overview and 400× for the microstructural details) and the crown-tooth interface was measured circumferentially in order to assess the adaptation quality. The milled crown restoration of the teeth is presented in the lower row of Figure 1.

Initial images at the prepared shoulder level were made and measured with the Inspect S SEM (FEI Company, Hillsboro, OR, USA) at the “Raluca Ripan” Institute of Chemistry and Research, Cluj-Napoca, Romania. Scanning electron microscopy of the samples was carried out at an acceleration voltage of 25 kV and low vacuum mode. Every tooth was analyzed, following the crown-tooth interface circumferentially under several magnifications.

The marginal adaptation was inspected with SEM at three points at a length of 2000 µm on each side: buccal, palatal, distal and mesial of the crown restored teeth, in good agreement with the literature [18]. The 2000 µm length was inspected at 100× magnification on the high resolution SEM images according to the literature [18,19,20]. These images were outputted on an A4 sheet format for an optimal view of the adaptation flaws. Each gap was measured and the marginal adaptation was expressed in percents according to the newest trend in the literature [24,25]. The morphological details of the gaps and flaw are presented at a high magnification of about 400× as well as the optimal marginal adaptation details. Samples were prepared in triplicate and the mean value of the marginal adaptation was calculated as the arithmetic average of three measurements.

The calculated mean values were statistically processed using the ANOVA test followed by the Tukey post hoc test at a significance level of *p* = 0.05. *p* values greater than significance level are situated in the same statistical group, while *p* < 0.05 presents significant statistical differences. The statistical analysis was performed with Origin Lab 9.8b 2019 software, Microcal Software Inc., Northampton, MA, USA.

## 3. Results

**Figure 2:**
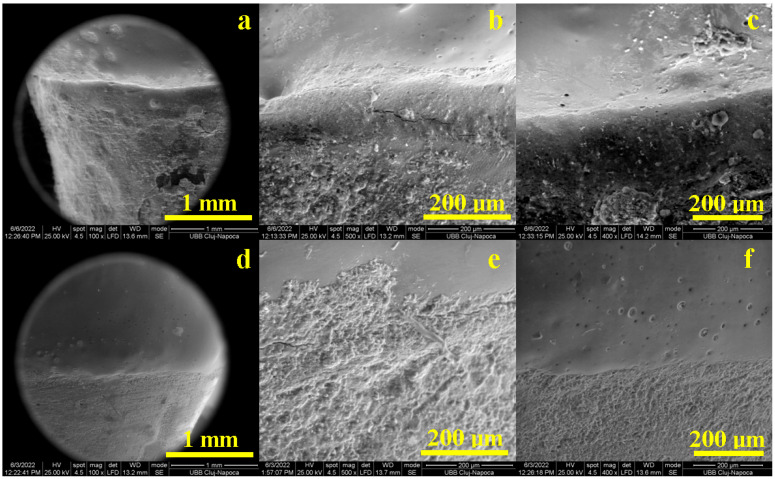
SEM images of the marginal adaptation on the buccal side for printed crowns: (**a**) general view, (**b**) defects within bonding layer and (**c**) flawless bonding layer and for milled crowns: (**d**) general view, (**e**) defects within bonding layer and (**f**) flawless bonding layer.

The SEM results of the marginal adaptation of the buccal side are presented in Figure 2: crown material is situated on the upper side of the images and tooth is on the lower side of the images. Printed-pressed crowns are well attached to the tooth surface through the cement layer, as seen in Figure 1. This high resolution SEM image was inspected at an A4 sheet format size and flaws areas were properly measured, resulting in a cumulative length of 120 µm which is tolerable according to [9,10,11], at the limit according to [12,13] and far exceeded according to [14]. The flaws’ nature was investigated at high magnification. Figure 2b reveals fine cracks with a thickness of about 1–3 µm involved on the cement layer as a consequence of its internal failure under the tangential forces induced by local miss adaptation of the crown to the teeth surface during the polymerization process. The well-adapted and cemented areas are evidenced in Figure 2c. It reveals perfect adhesion on both interfaces to the crown and tooth surface and an optimal compactness of the cement layer having a thickness of 125–150 µm.

The buccal side marginal adaptation of milled crowns reveals a uniform and continuous adhesion layer (Figure 2d). The high resolution inspection reveals a cumulative flaw length of 110 µm, which is very good according to [9,10,11] and poor according [14]. However, the flaw nature is like fine and minor cracks with a thickness of about 1–2 µm and local lengths of 30–50 µm (Figure 2e). Most of the cement layer having a thickness of 150 µm assures a compact bonding of both crown and tooth interfaces, assuring proper sealing and marginal adaptation (Figure 2f).

**Figure 3:**
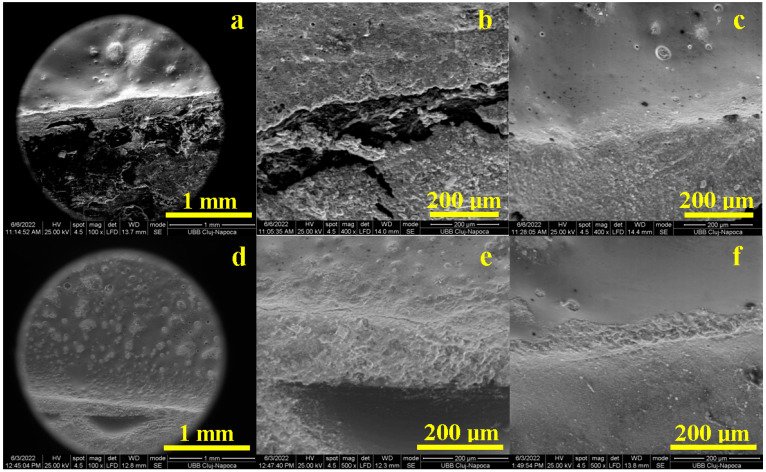
SEM images of the marginal adaptation on the palatal side for printed-pressed crowns: (**a**) general view, (**b**) defects within bonding layer and (**c**) flawless bonding layer and for milled crowns: (**d**) general view, (**e**) defects within bonding layer and (**f**) flawless bonding layer.

SEM images obtained for the palatal side are presented in Figure 3; the crown material is situated on the top and the tooth is on the bottom of these images. Printed-pressed crowns present a sharp cementation interface, Figure 3a, with some well-defined punctual flaws having a cumulative length of 180 µm. These flaws consist of successive gaps with a length of about 50 µm, creating local complete discontinuities within the cementation layer (Figure 3b). These gaps are generated by an insufficient filling of the crown-teeth interface by the cement due to local marginal miss-adaptation. The optimal cemented areas within the buccal marginal adaptation of printed-pressed crowns are illustrated by the image in Figure 3c. The cement layer is compact in its whole thickness of about 150 µm and perfectly adhered to both interfaces with the crown and tooth surface.

Milled crowns prove to be more adapted on the palatal side than printed-pressed ones, as seen in Figure 3d. The adhesion layer is well structured and very compact. Only a few small and fine cracks occur, having a cumulative length of 140 µm. These flaws morphology is better observed at high magnification in Figure 3e: fine cracks of about 1–3 µm thick and lengths of 20–40 µm. The flawless marginal adaptation aspect is presented in Figure 3f. The cement layer thickness varies from about 50 µm to 100 µm, depending on the local topography of the milled crown surface. It is very compact and assures optimal adhesion on both crown and tooth surfaces.

The distal side of the tooth is a difficult approach for marginal adaptation. It strongly depends on the crown finishing that influences contact with the cement layer, but the tooth surface also plays a key role in marginal adaptation success.

**Figure 4:**
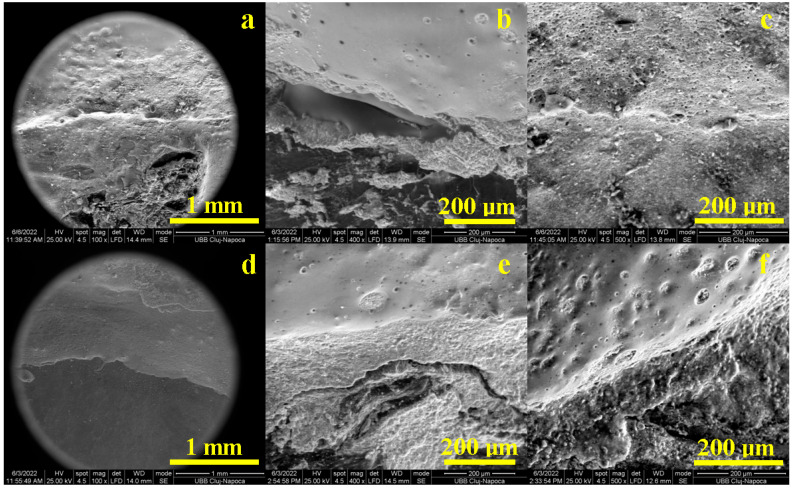
SEM images of the marginal adaptation on the distal side for printed crowns: (**a**) general view, (**b**) defects within bonding layer and (**c**) flawless bonding layer and for milled crowns: (**d**) general view, (**e**) defects within bonding layer and (**f**) flawless bonding layer.

Printed-pressed crowns present some irregularities on the distal side along with some topographical asperities on the tooth side that influence the cohesion within the cementation layer (Figure 4a). The high magnification reveals that the tooth surface has several irregularities on the distal side that affect the cement layer cohesion (Figure 4b). Thus, many voids appear between the tooth and crown, increasing the cumulative flaw length at about 350 µm. Most of the distal side features an optimal cementation, as revealed in Figure 4c, having a compact adhesion layer of about 50 µm, assuring proper adhesion on the crown and tooth surfaces.

Tooth irregularities occurring on the distal side affect the milled crown adaptation as well. Figure 4d shows that the cumulative flaw length is situated around 280 µm. The microstructural detail in Figure 4e reveals a fractional crack development on the tooth surface causing the cement layer local failure, while the crown side is perfectly bonded by the cement layer. Beside these local areas, the distal side marginal adaptation is well assured through the cement layer, as seen in Figure 4f.

**Figure 5:**
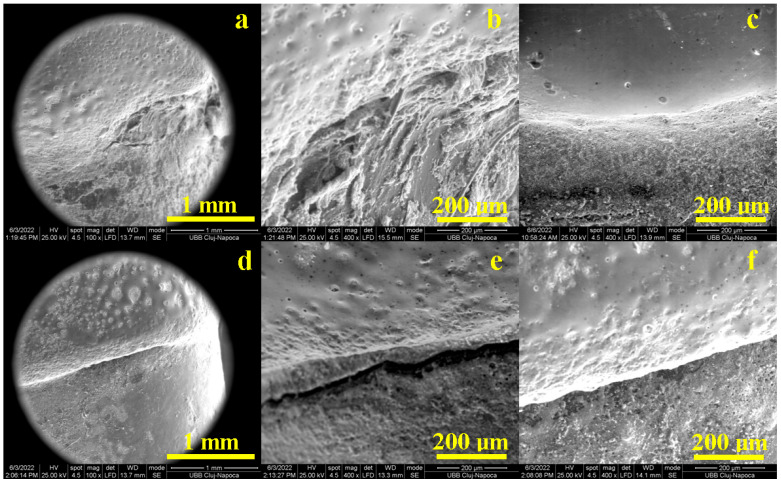
SEM images of the marginal adaptation on the mesial side for printed crowns: (**a**) general view, (**b**) defects within bonding layer and (**c**) flawless bonding layer and for milled crowns: (**d**) general view, (**e**) defects within bonding layer and (**f**) flawless bonding layer.

The mesial side of the tooth presents similar difficulties as distal. Figure 5a shows the marginal adaptation of the printed-pressed crowns. It reveals a cumulative flaw length of 380 µm that are distributed in some successive minor gaps (e.g., 30–75 µm length) occurring at the tooth-cement interface (Figure 5b). These are generated by local irregularities of the tooth surface. The smoothest tooth mesial surface assures optimal marginal adaptation, as observed in Figure 5c. The cement layer is very compact and adhered on both sides to the crown and to the tooth surfaces.

Thus, Figure 5e shows a major ditch within the cement layer containing several circular gaps of about 20–30 μm on its bottom, leaving the rest of the cement layer intact, assuring proper cohesion between interfaces.

**Figure 6:**
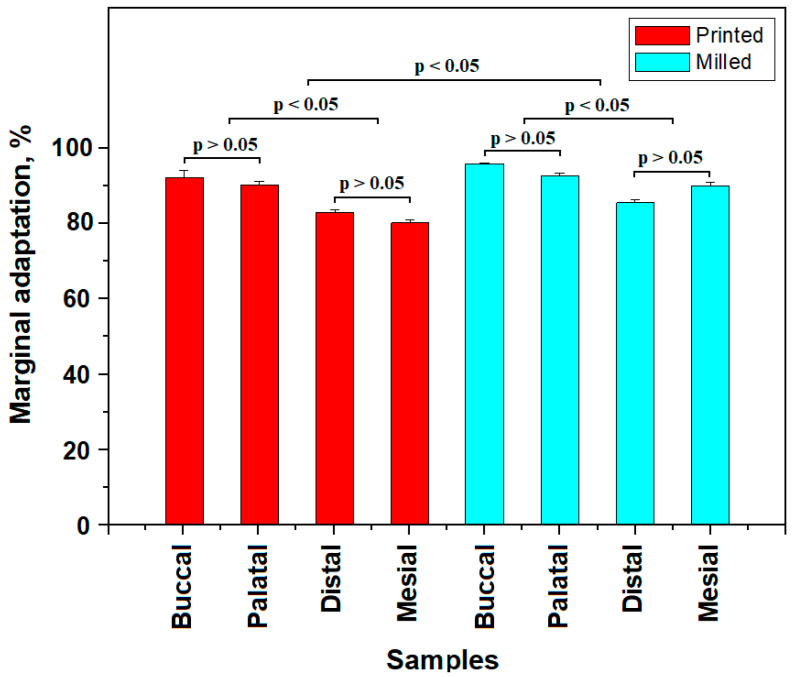
Statistical analysis of the marginal adaptation results, *p* > 0.05 means that there are no statistical differences and *p* < 0.05 shows significant statistical differences.

Finally, the cumulative gap length is about 220 µm. Significant portions of the marginal adaptation with compact and dense cement layer without median depression were also observed (Figure 5f). Overall, the milled crowns proved to be well adapted on the mesial side of the tooth. The mean values of marginal adaptation are presented in Figure 6.

All measurements effectuated on SEM images were centralized and processed to express the percentage of marginal adaptation according to the actual trends [24,25]. The obtained values are centralized and displayed in Figure 6, along with the statistical analysis results. 

## 4. Discussion

The printed-pressed crowns have two statistical populations: the first one has marginal adaptation in the range of 90.30–91.93% corresponding to the palatal and buccal sides and the second statistical population has marginal adaptation in the range of 80.20–82.50% corresponding to the mesial and distal sides. This proves that the surface conditioning difficulties regarding the mesial and distal sides cause a statistically significant decrease in marginal adaptation.

Milled crowns also present two statistical populations. The first one is represented by the higher values of the marginal adaptation situated in the range of 92.30–94.83% corresponding to the palatal and buccal sides. The second statistical population is represented by the lower values of the marginal adaptation situated in the range of 86.36–90.00% corresponding to the distal and mesial sides, respectively. The distal and mesial sides’ difficulties regarding surface conditioning also affect marginal adaptation.

The second step of the statistical analysis was the comparison of the statistical population of printed-pressed crowns with those belonging to the milled crowns. Significant statistical differences were found (*p* < 0.05), proving that milled crowns have better marginal adaptation than printed-pressed crowns on all investigated tooth sides.

Resistance to fracture and biocompatibility are two predictors of a prosthetic restoration, while the other two are aesthetics and marginal adaptation [26]. In the modern world, the desire for aesthetics has expanded massively, which has stimulated the dental industry to simultaneously increase the aesthetic and mechanical properties of restorative materials. In this regard, dental ceramics, through their excellent aesthetical properties, high biocompatibility and reliability, gain important ground, including in prosthetic restorations in the posterior areas of the dental arches. Here, after the primary position was successfully occupied by monolithic zirconia [27] an increasing extensive insertion of monolithic LD ceramics can be observed, argued by the good long-term results after 8.7–11 years with reported survival rates of 83.5–98.2% [28,29,30], and not only.

The marginal adaptation of restorations depends on several parameters, such as preparation technique, restoration material, manufacturing process, tooth location, cementation procedure, etc. Additionally, marginal adaptation of CAD-CAM restorations is considered to be influenced by fabrication stage, number of units in substructure, material stiffness, type and thickness of the luting cement, type of CAM system and CAD-CAM software parameter settings [31,32,33]. In light of the challenges of digital technologies in dentistry, our in vitro research focused on the marginal adaptation of LD crowns manufactured by two different methods and cemented on extracted human teeth of posterior areas of the jaw (premolars). Our results showed that the different processing methods of dental crowns (CAD-CAM milling or 3D-printing) have an impact on the marginal adaptation of these all-ceramic prosthetic restorations; thus, we rejected the null hypothesis. The printed-pressed crowns have the marginal adaptation in the range of 90.30–91.93%, corresponding to oral and buccal sides. It reveals that the surface conditioning difficulties regarding the proximal areas cause a statistically significant decrease in marginal adaptation. Crowns produced by CAD-CAM milling have had higher values of marginal adaptation, situated in the range of 92.30–94.83%, corresponding to the buccal and oral sides. The distal and mesial sides’ difficulties regarding surface conditioning also affect marginal adaptation.

Each fabrication method presents strengths and weaknesses that need to be considered regarding the quality of the marginal adaptation of the manufactured restorations. CAD-CAM technology has several advantages over conventional methods in terms of quality, speed and ease of use. Chou et al. [34] experimentally demonstrated the superior marginal adaptation of metal crowns fabricated using CAD-CAM digital methods than that of the traditionally manufactured ones. In 2023, Ferrini et al. [18,35] showed that among the metal-free prosthetic restorations obtained by CAD-CAM technology such as zirconia, composite and LD, the zirconia crowns showed the best precision of fit at the preparation margins, but all these materials showed clinically acceptable marginal gaps. This could be explained by different factors, such as the fact that dental CAD-CAM systems were developed for processing polycrystalline materials, which led to the creation of more accurate results. Their study included the newest generations of milled dental ceramics, such as those made of LD, from various manufacturers. Thus, it was concluded that although the mean marginal gap of the IPS E-Max milling crowns (Ivoclar Vivadent AG, Schaan, Liechtenstein) of 49.2 µm was statistically significantly greater than those of all others, this threshold is among the clinically acceptable mean maximum gap of less than 120 μm [36,37].

Regarding LD restorations we also focused on in our study, it has to be mentioned that along with CAD-CAM technology, these can be either manufactured with the conventional heat-pressed method. This factor also does not seem to significantly influence the values reported for the marginal adaptation of the LD crowns manufactured by both methods. A recent large meta-analysis was conducted by Sanches et al. [38] thus reported insignificant differences regarding the marginal adaptation of the LD crowns manufactured with CAD-CAM and heat-pressed, a convergent result with that of Son et al. [39], which determined similar values regarding the marginal discrepancy (132.2 μm for the CAD-CAM group and 130.2 μm for the conventional pressed technique group). The buccal side marginal adaptation of milled crowns revealed a uniform and continuous marginal adaptation. The high resolution inspection reveals a cumulative flaw length of 110 µm which is very good. The mean marginal gap of the milling crown was statistically significantly greater than those printed-pressed.

Extensive debates among clinicians also took place regarding the role of different marginal finish line configurations on the marginal adaptation of all ceramic-crowns, also including those of LS. Faruqi et al. [40] concluded that the chamfer finish lines produced better fitting restorations, and heat-pressed LD crowns showed better adaptation at the margins than both layered zirconia and monolithic zirconia. However, a micro-CT analysis of LD crowns made exclusively by CAD-CAM technology (IPS E-Max CAD, Ivoclar AG, Schaan, Liechtenstein) based on 3 different finish lines (rounded shoulder, chamfer, feather-edge), shows that the restorations with a rounded shoulder finish line had the best marginal fit, while the milling LD crowns with a feather-edge finish line had the best internal fit but the poorest marginal fit, Rizonaki et al. demonstrate that the combination of feather-edge and LD crowns does not meet the requirements for providing reliable restorations [41]. In our study in which rounded shoulder finish line preparations were performed, the values were convergent/divergent compared to the mean value reported of 87.6 µm for lithium disilicate crowns from the same manufacturer (E-Max, Ivoclar, Vivadent AG, Schaan, Liechtenstein) also evaluated by SEM, but with chamfer finish line preparation [42].

Marginal adaptation of all-ceramic restorations is also sensitive to the physical and chemical properties of luting adhesive resins [43] and to clinicians’ cementation procedures (finger pressure or standardized loading during cementation) [44]. In their micro-CT evaluation of marginal adaptation of LD ceramic crowns cemented with three different resin cements. Peroz et al. determined clinically not acceptable marginal adaptation by using Variolink II cement, the authors attributed this inconvenience to the three-step protocol required (priming, adhesive, bonding) but especially to the bonding agent, as well as the use of hydrofluoric acid during the cementation process, which results in the formation of micromechanical voids [44] it is noteworthy that we also used the same resin cement in our present research.

Few studies are available on the marginal adaptation of 3D printed LD crowns, as it is still a pioneer technology. Data acquisition and processing follow the same steps as the conventional CAD/CAM system, but the subtractive method by milling a ceramic block is replaced with the additive one [45]. Thus, the significant amount of waste that is produced upon milling is avoided in the case of the 3D printing promising technology and the 3D-printed polymer-based restoration is processed using the press technique to gain the final restoration [46]. A micro-CT comparison of the adaptation of CAD/CAM and 3D-printing/pressed LD monolithic crowns by Ottoni et al. [47] revealed a larger gap thickness of 3D-printing/pressed at the axial angle and axial wall and smaller at the occlusal region in comparison to the milled LD crowns.

The newest trends are related to the light curing method. Some authors suggest that different curing methods (with pre-curing/without pre-curing could influence micro-leakage and the marginal adaptation. It is important to decide when the adhesive should be polymerized in relation to the application of cement material. For the clinician, it is difficult to ascertain if it is necessary to polymerize before applying the cement material or leave the adhesive un-polymerized. It should be noted that a thicker polymerized layer of adhesive can determine an improper position of the crown on the finish line, which could lead to a marginal gap [48,49]. Analysis of these findings suggests that pre-curing the adhesive results in a thicker adhesive film. Hence, the pre-cured adhesives showed the best resistance to penetration, although the film thickness of these luting agents was only slightly increased. On the other hand, for adhesives that were not pre-cured before their use, no adhesive film could be distinguished from the resin-luting layer. This technique is not recommended for restorations with reduced retention, such as crowns with low heights. The crowns require a strong bond to the tooth structure to ensure their longevity and prevent discoloration.

## 5. Conclusions

The marginal adaptation of the crowns strongly depends on the surface conditioning. The printing process combined with pressing proves to be less precise than the milling of the crowns. Thus, it was observed that the milled crowns presented better marginal adaptations than printed-pressed ones. This indicates that the milling process allows a better fit of the crown to the tooth surface and preserves the integrity of the bonding cement layer.

## Figures and Tables

**Table 1:** Materials description.

Material Name	Manufacturer	Composition	Lot Number
Lithium disilicate	(Ivoclar Vivadent AG, Schaan, Liechtenstein)	SiO_2_-Li_2_O-Al_2_O_3_-K_2_O-ZrO_2_-P_2_O_5_	U51702
Variolink Esthetic DC	(Ivoclar Vivadent AG, Schaan, Liechtenstein)	ytterbium trifluoride, urethane dimethacrylate, 1,10-decandiol dimethacrylate, α,α -dimethylbenzylhydroperoxide	X54712
Adhese universal	(Ivoclar Vivadent AG, Schaan, Liechtenstein)	2-hydroxyethyl,methacrylate, ethanol,1,10-decandiol dimethacrylate, campherquinone, 2-dimethylaminoethyl methacrylate	Z04JTK

## Data Availability

Data are available on request from the corresponding author.
